# Core-Shell Nanoencapsulation of α-Tocopherol by Blending Sodium Oleate and Rebaudioside A: Preparation, Characterization, and Antioxidant Activity

**DOI:** 10.3390/molecules23123183

**Published:** 2018-12-03

**Authors:** Junbo He, Hao Shi, Shuangshuang Huang, Lijuan Han, Weinong Zhang, Qixin Zhong

**Affiliations:** 1 Key Laboratory for Deep Processing of Major Grain and Oil, Ministry of Education, College of Food Science & Engineering, Wuhan Polytechnic University, Wuhan 430023, China; junb112he@whpu.edu.cn (J.H.); go_live@yeah.net (H.S.); Huangss0828@163.com (S.H.); hanlj.whpu@hotmail.com (L.H.); 2 Hubei Key Laboratory for Processing and Transformation of Agricultural Products, Wuhan Polytechnic University, Wuhan 430023, China; 3 Department of Food Science, The University of Tennessee, Knoxville, TN 37996, USA; qzhong@utk.edu

**Keywords:** core-shell nanoencapsulation, α-tocopherol, sodium oleate, rebaudioside A, antioxidant activity

## Abstract

Nanoencapsulation of α-tocopherol (α-TOC) by blending sodium oleate (NaOl) and rebaudioside A (RebA) was successfully prepared by self-assembly method under mild conditions. The optimized nanoemulsion showed the loading capacity of α-TOC was 30 wt% of sodium oleate. FTIR analysis suggested that hydrogen bonds and hydrophobic interactions were the major forces in α-TOC-NaOl/RebA complexes that were spherical and possessed well-distinguishable core-shell structures. The freeze-dried α-TOC-NaOl/RebA complexes had great stability under ambient conditions. The release profile of α-TOC showed a first-order kinetics reaching around 67.9% after 90 h at 25 °C. Nanoencapsulation improved dispersibility and greatly increased the antioxidant activity of α-TOC. Therefore, the stable α-TOC-NaOl/RebA core-shell complexes prepared from “generally recognized as safe” (GRAS) ingredients have great potential to supplement α-TOC in food and cosmetic products.

## 1. Introduction

In recent years, there has been an increasing demand for food and cosmetic products to provide bio-functional ingredients to promote the health and wellbeing of consumers [1]. Lipophilic vitamin E, a group of well-known antioxidants [2], has various biological functions for human health, such as acting as signaling and gene regulation molecules [3,4] and preventing age-related and chronic diseases [5,6]. Therefore, much work has been done to incorporate vitamin E in functional foods, beverages, cosmetics, and pharmaceutical products.

The vitamin E family contains four tocopherols (α, β, γ, and δ) and four corresponding tocotrienols (α, β, γ, and δ), of which α-tocopherol (α-TOC) displays the highest biological activity [7]. To incorporate α-TOC in various products, many challenges are to be overcome during processing and storage, such as very low water-solubility and instability [8,9]. A practical way is to encapsulate α-TOC in a colloidal delivery system that is then incorporated in food, cosmetic, or pharmaceutical products. For transparent products, nanoscale delivery systems are needed to preserve the appearance during shelf-life storage.

Various methods of encapsulating α-TOC have been reported [10,11,12,13,14,15,16,17,18], such as edible orange oil-in-water emulsions [19], polycaprolactone nanocapsules [20], injectable hydrogels prepared using PEG/α-TOC copolymer [21], microcapsules of zein and β-cyclodextrin [22], and zein/chitosan complexes [23]. Lipid-based core-shell nanoparticles are a promising delivery system for small molecules [24], using lipids as the shell to coat the core with charged small molecules. Charged lipids have been reported to design core-shell nanoparticles, such as zwitterionic lecithin [25], cationic 1,2-dioleoyl-3-trimethylammonium-propane (DOTAP) [26], and anionic phospholipid dioleoylphosphatidic acid (DOPA) [27]. Much work is needed to study lipids derived from natural raw materials to prepare core-shell nanoparticles so as to utilize their biodegradability, low toxicity, and surfactant properties [28].

Sodium oleate (NaOl) derived from natural plant oils is approved for direct addition in foods by the US Food and Drug administration [29] and European committee [30]. NaOl has been studied as a carrier material of lipophilic compounds. However, it has low solubility in cold water or neutral aqueous systems as well as insolubility in hard water. These properties limit the development of desirable delivery systems [31]. Recently, rebaudioside A (RebA), a steviol glycoside, was reported to function as a co-surfactant to stabilize NaOl solution even at neutral pH [31]. Therefore, we hypothesize that NaOl, an anionic lipid, can interact with RebA to form a stable shell to encapsulate α-TOC as the core.

**Figure 1:**
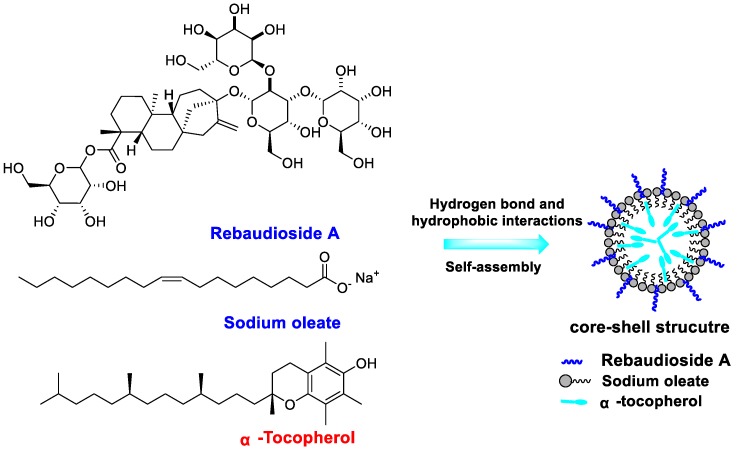
Schematic illustration of the formation of NaOl/RebA complex for the encapsulation of α-TOC.

Herein, we reported core-shell nanoparticles of α-TOC coated with NaOl/RebA blend for the first time (Figure 1). The optimized formulation was obtained by evaluating the particle size, polydispersity index (PDI), and zeta potential. The loading capacity, storage stability, Fourier transform infrared (FTIR) spectroscopy, particle morphology, release profile, and antioxidant activity were also investigated.

## 2. Results and Discussion

### 2.1. Preparation and Characterization of α-TOC Loaded Nanoemulsions

It was reported the mass ratio of NaOl and RebA plays an important role in the stability of mixtures [31]. Therefore, the influence of the mass ratio of NaOl and RebA was first studied. The system containing 1 wt.% NaOl and 1 wt.% RebA had the best stability (not shown), with particle size, PDI, and zeta potential of 149 ± 3 nm, 0.12 ± 0.02, and −80.6 ± 2.4 mV, respectively. The results differ slightly from a study [31] that reported a stable mixture with 1 wt.% NaOl and 0.8 wt.% RebA. The difference may be due to the purity of our NaOl being higher than 82%, as it was in the literature study.

**Figure 2:**
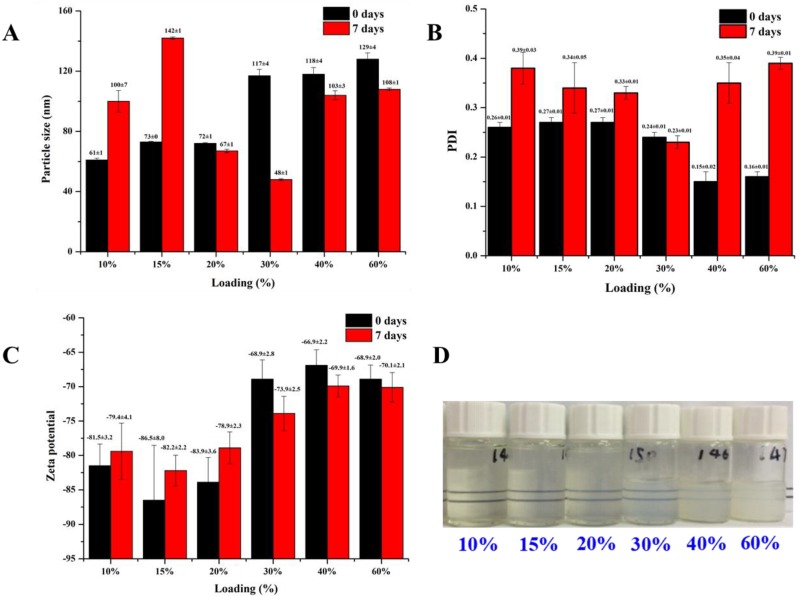
Effects of α-TOC loading, in percentages of sodium oleate mass, on (**A**) particle size, (**B**) polydispersity index (PDI), (**C**) zeta potential before and after seven-day ambient storage at 25 °C, as well as (**D**) appearance of fresh dispersions. Error bars are standard deviations (*n* = 3).

Subsequently, different amounts of α-TOC, presented with the percentage to NaOl mass hereafter, were encapsulated in the mixture with 1 wt.% NaOl and 1 wt.% RebA. From the data depicted in Figure 2A, particle sizes of fresh α-TOC-containing nanoemulsions increased with an increase in α-TOC content, which agreed with increases in visual turbidity (Figure 2D), whereas the PDI decreased (Figure 2B). After seven-day storage, the treatments with α-TOC at 10% and 15% masses of NaOl showed the increase in particle size, whereas other samples showed the decreased particle size. Figure 2B shows the PDI values of most samples increased to above 0.3 after seven-day storage. This suggests the possibility that changes in particle sizes were caused by structural rearrangement after storage. The treatments with α-TOC at 40% and 60% masses of NaOl showed visible phase separation after seven-day storage, while other samples remained visually stable. This indicates the 40% and 60% loading of α-TOC exceeded the maximum loading that can be stably encapsulated in the hydrophobic inner core. Fresh dispersions had a zeta-potential all over 60 mV (Figure 2C), which is typically sufficient to prevent aggregation of colloidal particles by electrostatic repulsion [32], and particle PDI change likely due to structural rearrangement of α-TOC during storage, which may cause instability. Therefore, the maximum loading of α-TOC at the studied conditions was about 30% mass of NaOl, which was then used in the rest of this study. Furthermore, the encapsulation efficiency value was measured to be 98.14 ± 0.37%. The high encapsulation efficiency of α-TOC is believed to be due to its oily property, which keeps almost all the α-TOC in the hydrophobic inner core.

### 2.2. Stability of α-TOC in the Freeze-Dried Samples

**Figure 3:**
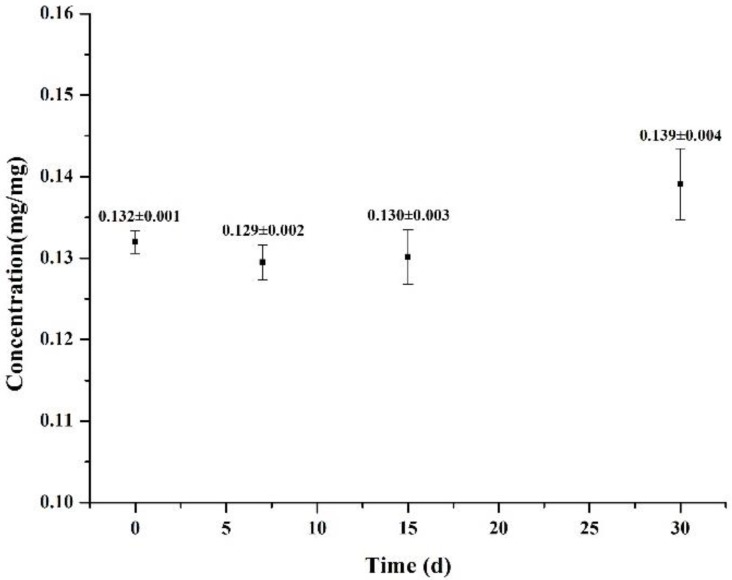
The stability of α-TOC in freeze-dried nanoparticles during 30 days storage. The unit mg/mg means the amount of α-TOC per milligram of freeze-dried α-TOC-NaOl/RebA complexes. Error bars are standard deviations (*n* = 3).

The stability of α-TOC was investigated after storage of a freeze-dried sample in a desiccator for up to 30 days at 25 °C. The theoretic concentration of α-TOC in the freeze-dried sample was 0.130 mg/mg, which agreed with the quantified α-TOC content that did not change significantly during 30-day storage (*p* > 0.05; Figure 3). Therefore, encapsulation, freeze-drying, and desiccator storage resulted in good storage stability of α-TOC.

### 2.3. Interaction Forces Studied With FTIR

**Figure 4:**
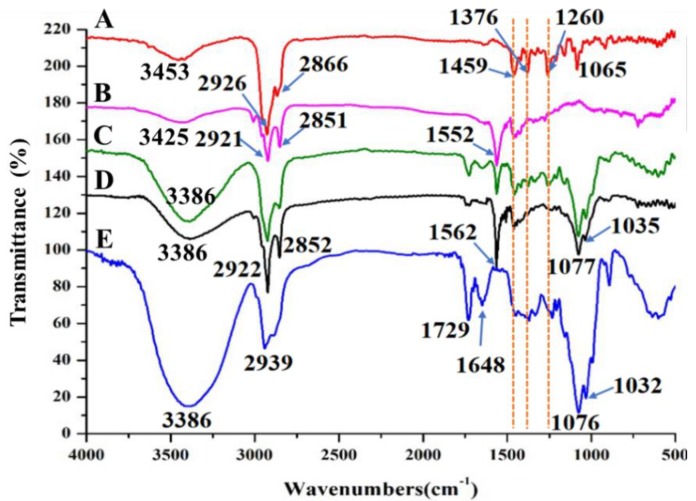
FTIR spectra of individual ingredients and their complex samples: (A) α-tocopherol, (B) NaOl, (C) simple physical mixture of α-tocopherol, NaOl, and RebA, (D) α-TOC-NaOl/RebA complex, and (E) RebA.

FTIR was used to characterize intermolecular interactions of α-TOC-NaOl/RebA complexes. The FTIR spectra of individual compounds, i.e., α-TOC, NaOl, RebA, their physical mixture, and α-TOC-NaOl/RebA complexes, are presented in Figure 4. Significant hydrogen bonds were found in the spectra of NaOl and RebA, indicated by bands at 3452 cm^−1^ and 3386 cm^−1^, respectively [33]. For the physical mixture and freeze-dried complexes, the peak corresponding to hydrogen bonds shifted to 3386 cm^−1^. Hydrogen bonds can be formed between carboxyl and hydroxyl groups; thus, intermolecular hydrogen bonds can be easily formed between NaOl and RebA (Figure 1). The formation of strong hydrogen bonds between NaOl and RebA is in accordance with the literature [31].

The α-TOC spectrum showed bands at 2926 cm^−1^ and 2866 cm^−1^ (asymmetric and symmetric stretching vibration of the CH_2_), 1459 cm^−1^ (phenyl skeletal), 1376 cm^-1^ (methyl symmetric bending), 1260 cm^−1^ (CH_2_), and 1065 cm^−1^ (plane bending of phenyl stretching) [34]. In the physical mixture, all these characteristic absorption peaks of α-TOC were found. Conversely, several major changes were observed in the spectrum α-TOC-NaOl/RebA complexes. The disappearance of three absorption peaks of α-TOC at 1459 cm^−1^, 1376 cm^−1^, and 1260 cm^−1^ indicate the encapsulation of α-TOC in the core of complexes. The hydrophobic attraction was thought to be the force involved in the self-assembly of α-TOC-NaOl/RebA complexes.

### 2.4. Particle Structures Studied with TEM

**Figure 5:**
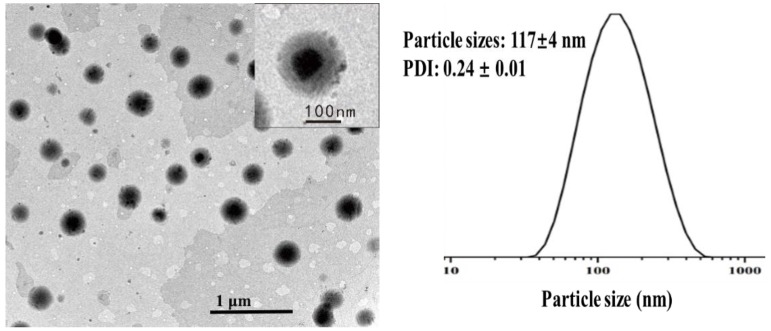
Transmission electron microscopy image of α-TOC-NaOl/RebA complex with 30% loading of α-TOC.

The morphology of nanoparticles was observed using TEM. As shown in Figure 5, most particles were spherical and had variations in the dimension. An enlarged view of a single particle showed well-distinguishable core-shell structure. As α-TOC is not charged but the α-TOC-NaOl/RebA complex particles are highly negatively charged (Figure 2C), it is likely that the shell is composed as NaOl/RebA that can easily form complexes [31], while the core is nonionic α-TOC. The core-shell structures may have enabled a good protection effect for α-TOC during storage (Figure 3).

### 2.5. Release Profile of α-TOC from Nanoemulsion

**Figure 6:**
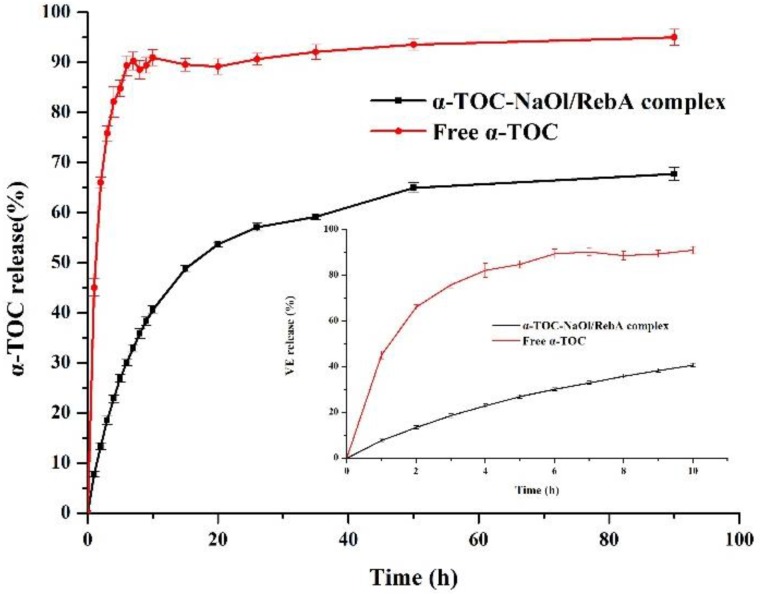
Release profile detected for α-TOC in the reservoir for dialysis bags containing α-TOC-NaOl/RebA nanocomplex dispersions and free α-TOC in PBS medium containing 20% ethanol and 0.5% Tween 80. The inset figure shows release kinetics in the first 10 h. Error bars are standard deviations (*n* = 3).

The release kinetics of α-TOC at the studied conditions are shown in Figure 6. The phosphate buffer (pH 7.2) with 20% ethanol and 0.5% Tween-80 was used to provide the sink condition. The treatment of free α-TOC showed a quick increase to 90.5% in 7 h release, which reflects the diffusion of α-TOC from the dialysis back to the outer sink. This was followed by a much smaller increase rate in the subsequent hours to achieve the highest release of 95.2% (Figure 6). In contrast, the release rate of α-TOC from NaOl/RebA core-shell nanoparticles was much lower than free α-TOC. After 90 h, approximately 67.9% of α-TOC was released from the nanoemulsion, which is lower than the free α-TOC with 95.2% release. The slow release profile of α-TOC from nanoparticles demonstrates the preferred partition of α-TOC in nanoparticles with hydrophobic NaOl/RebA complexes rather than the continuous phase with 20% ethanol and 0.5% Tween 80.

The in vitro release equations of α-TOC from nanoemulsion were listed in Table 1. The data revealed the sustained release profile of α-TOC fitted best to Weibull equation. The release behavior of the encapsulated drug could be affected by the encapsulation pattern and surface properties [35]. Based on the observed release profile and Weibull equation, it could be deduced that the α-TOC-NaOl/RebA complex was a core-shell structure, and α-TOC was enriched in the core. The α-TOC incorporated in the shell released rapidly, and α-TOC loaded in the core could only release slowly by means of dissolution and diffusion [36].

### 2.6. Antioxidant Activity of Nanoemulsion

**Figure 7:**
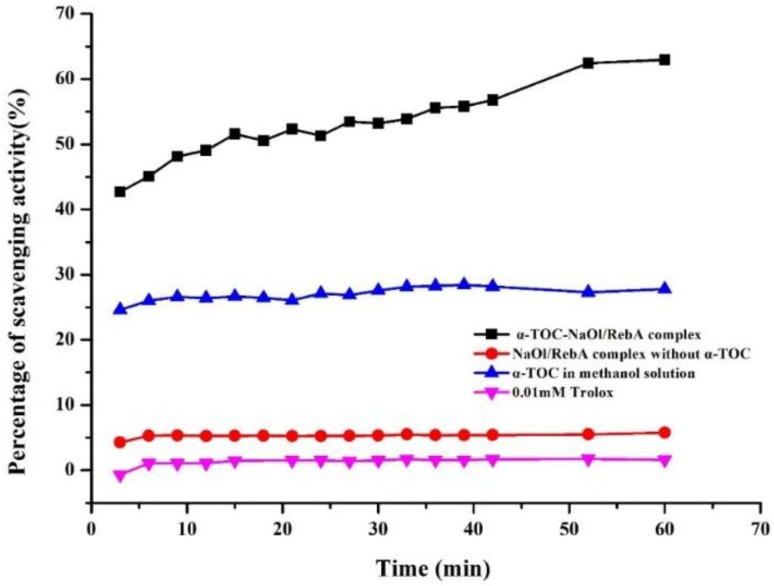
Antioxidant activity of α-TOC-NaOl/RebA complex (concentration of α-TOC was 0.19 mg/mL) assayed for percentage of scavenging activity using ABTS radical scavenging assay, in comparison for same amounts of free α-TOC pre-dissolved in methanol and NaOl/RebA complex without α-TOC.

α-TOC is a well-known lipophilic antioxidant that can prevent lipid peroxidation. However, its bioactivity is largely limited because of its low water solubility and stability. Several previous studies demonstrated that the encapsulation of α-TOC in a lipophilic matrix is necessary to enhance its antioxidant activity [22,37,38]. In the present study, ABTS radical scavenging assay was used to determine the antioxidant activity. The ABTS scavenging activity was evaluated for α-TOC-NaOl/RebA complex, NaOl/RebA complex without α-TOC, α-TOC in methanol solution, and 0.01 mM Trolox solution. The results are shown in Figure 7. The kinetic pattern observed for the α-TOC-NaOl/RebA complex showed a much faster initial scavenging activity (antioxidation) step, followed by a slower step. In contrast, other treatments showed almost no difference in the percentage of scavenging activity during the testing. Most importantly, the ABTS scavenging activity of the α-TOC-NaOl/RebA complex was double that of α-TOC in methanol solution. The Trolox equivalent antioxidant capacity (TEAC) values of α-TOC-NaOl/RebA complex was 2.31 mmol/L, almost twice the value of α-TOC in the methanol solution of 1.16 mmol/L. The difference could be explained by the improved dispersibility of encapsulated α-TOC as well as the large surface of nanoparticles, which together facilitated the reaction kinetics of α-TOC with free radicals in the aqueous phase. Similar observations have been reported on the improvement of antioxidant activity after dispersion in colloidal particles [39,40]. Our results demonstrated that the NaOl/RebA nanoparticles could be superior nanoencapsulation matrix to enhance the antioxidant activity of α-TOC.

## 3. Materials and Methods

### 3.1. Materials

NaOl (>97% purity), RebA (96% purity), and DL-α-TOC (96% purity) were purchased from Aladdin Co., Ltd. (Shanghai, China). 2,2-azino-bis(3-ethylbenzothiazoline-6-sulphonic acid) diammonium salt (ABTS) and Trolox were purchased from the Beyotime Biotechnology (Shanghai, China). All materials were used without further purification. Distilled or deionized water was used throughout the study.

### 3.2. Preparation of Nanoemulsions

NaOl and RebA were added at mass ratios of 2:1, 1:1, or 1:2 into deionized water and stirred at room temperature (25 °C) for 1 h. The mixtures were evaluated immediately and after 7 days for their particle size, PDI, and zeta potential to get the optimal mass ratio. On the basis of this, α-TOC with 10%, 15%, 20%, 30%, 40%, and 60% masses of NaOl was added into the NaOl/RebA nanoemulsion. The NaOl concentration in all experiments was kept at 1% (*w*/*v*) in deionized water (6 mL).

### 3.3. Characterization of Nanoemulsions

Particle size, PDI, and zeta potential measurements of nanoemulsion were performed using a Malvern Zetasizer Nano ZS90 Particle Analyzer (*λ* = 633 nm, material/dispersant RI 1.590/1.330). Samples were prepared by diluting a sample for eighty folds using double distilled water at pH 7.0. Each sample was repeated three times to obtain the mean value.

### 3.4. Encapsulation Efficiency of α-TOC

Based on the optimized loading efficiency studied above, the encapsulation efficiency of α-TOC was determined by ultrafiltration method using centrifugal filter tubes (Amicon Ultra-0.5, 10 kDa, Millipore, Billerica, MA, USA), as described in previous studies [41]. The percentage of encapsulated α-TOC was calculated by the difference between the total amount of α-TOC added in the nanoemulsion and the amount of α-TOC encapsulated. The amount of α-TOC encapsulated was calculated as the initial α-TOC minus the free α-TOC that remained in the filtrate aqueous phase after ultrafiltration by centrifugation (15 min, 5000× *g*) of the nanoemulsion. Analysis of α-TOC was performed by the HPLC method reported below. The encapsulation efficiency was calculated using the following equation:

EE(00)=(Wa−WsWa)×100%

where EE is the encapsulation efficiency and W_a_ and W_s_ are the total weight of α-TOC added and weight of free α-TOC, respectively.

### 3.5. Freeze-Drying

The nanoemulsion with optimized α-TOC loading was freeze-dried to evaluate its storage stability. Briefly, the freshly prepared nanoemulsion was frozen at −80 °C overnight and then freeze-dried at −60 °C/0.014 mBar for 24 h (model Alpha 1-2LD plus, German Christ Company, Osterode am Harz, Lower Saxony, Germany). The freeze-dried sample was stored in a desiccator at 25 °C before other experiments.

### 3.6. Stability of α-TOC in the Freeze-Dried Sample

The stability of α-TOC in the freeze-dried sample was evaluated after storage in a desiccator for 0, 7, 15, and 30 days. Briefly, 10 mg of freeze-dried sample was dissolved in 4 mL dry methanol and sonicated for 30 min, and then 500 µL of the solution was diluted by 10 times and used for HPLC detection (detection at 285 nm). A Waters 1525 series HPLC system (Waters Corporation, Milford, CT, USA) was used for the HPLC analysis. The chromatography was performed on an Agilent C18 column (4.6 × 150 mm, 5 μm). An isocratic program was applied with methanol at a flow rate of 1.0 mL/min and a column temperature of 25 °C. The detector response was linear from 0.01 to 1 mg/mL with a correlation coefficient of 0.9999. The concentration of α-TOC in the freeze-dried sample was calculated by dividing the amount of α-TOC quantified by HPLC with 10.

### 3.7. FTIR Spectra

The freeze-dried α-TOC-NaOl/RebA complex, pure α-TOC, NaOl, RebA, as well as their physical mixture were individually mixed with KBr and recorded in the wavenumber range of 4000–500 cm^−1^. Measurements were performed on a Thermo Nicolet NEXUS 670 FTIR Raman spectrometer (Thermo Nicolet Corporation, Madison, WI, USA) and were obtained with 16 scans.

### 3.8. Transmission Electron Microscopy (TEM)

The morphology of α-TOC-NaOl/RebA complexes was evaluated using a Hitachi H-7650 transmission electron microscopy equipment (Hitachi High-Technologies, Pleasanton, CA, USA). The nanoemulsion was initially diluted by 80 times with double distilled water and ultra-sounded for 15 s, deposited onto 200-mesh copper grids, and allowed to dry for 15 min. After drying, the sample was analyzed at a voltage of 80 kV. The bright-field TEM image was shown in Figure 5.

### 3.9. Release Profile of α-TOC

Lyophilized nanoparticles were dissolved in the release medium containing phosphate buffer saline (PBS, pH 7.2) that was added with ethanol (20%, *v*/*v*) and Tween-80 (0.5%, *w*/*v*) to increase α-TOC solubility, and the concentration of α-TOC was 1.00 mg/mL. The release studies were carried out using dialysis membrane bags with a molecular weight cut-off of 3500 Da (Yobios BioTech, Xi’an, China). The membrane bags were soaked in water overnight before use. The bags were filled with 10 mL of a dispersion containing 1.00 mg/mL of α-TOC, immersed in 400 mL release medium, and rotated at 100 rpm and 25 °C. A total of 10 mL of sample was withdrawn from the release medium at predetermined time intervals and the same amount of release medium was replenished. The samples were analyzed for the absorbance of 285 nm using a UV-visible spectrophotometer (Beckman Coulter, DU-730, Fullerton, CA, USA). The amount of α-TOC was then calculated by a calibration curve established with standard solutions of free α-TOC dissolved in the release medium (*R*^2^ = 0.9997). The release of free α-TOC was performed in the same manner as the concentration of 1.00 mg/mL dissolved in the release medium. All measurements were performed in three replicates.

### 3.10. Antioxidant Activity

The antioxidant activity of α-TOC-loaded NaOl/RebA complexes was evaluated using ABTS radical scavenging assay in a 96-well microplate as described in the literature [42]. Briefly, ABTS free radicals were prepared by mixing ABTS aqueous solution (7 mM) and potassium persulfate (4.95 mM), followed by incubating in the dark at room temperature for 12 h. The ABTS working solution was diluted with PBS (0.2 M, pH 7.4) to a certain concentration to reach the absorbance value of about 0.7 at 734 nm. Then, 10 μL of a nanoemulsion sample diluted by 16 times to make the concentration of α-TOC being 0.19 mg/mL was added to 200 μL of ABTS working solution and reacted for 6 min in the dark at room temperature. The absorbance at 734 nm was recorded with an automated microplate reader (PerkinElmer EnSpire, Waltham, MA, USA) at 25 °C. A control nanoemulsion without α-TOC and a free α-TOC solution pre-dissolved at 0.19 mg/mL in methanol were assayed for comparison. The ABTS radical scavenging activity of the sample was calculated as follows:

radical scavenging activity (%) = [(A1 − A2)/A1] × 100%

where A1 is the absorbance of the control (ABTS solution without test sample) and A2 is the absorbance in the present of the test samples.

### 3.11. Statistical Analysis

The data reported in this paper were presented as mean ± standard deviation (SD). Significance of differences was evaluated using Student’s *t*-test with *p* < 0.05.

## 4. Conclusions

In the present study, α-TOC-NaOl/RebA core-shell nanoparticles were successfully prepared under mild conditions. The 30% loading capacity and 98.14 ± 0.37% encapsulation efficiency demonstrated that it is a promising approach to encapsulate α-TOC. Physicochemical analyses suggested that electrostatic interaction, hydrogen bond, and hydrophobic interaction are the main forces in the formation of α-TOC-NaOl/RebA nanocomplexes. The inclusion of α-TOC in nanoparticle core resulted in the stability of freeze-dried α-TOC-NaOl/RebA complex during ambient storage in a desiccator. Nanoencapsulation resulted in the first-order release kinetics of encapsulated α-TOC to amount to 67.9% after 90 h of incubation at 25 °C. Dispersion of lipophilic α-TOC in nanoparticles and the enormous surface area of nanoparticles enabled the much-improved antioxidant activity. Therefore, the stable α-TOC-NaOl/RebA core-shell nanoparticles prepared from “generally recognized as safe” (GRAS) ingredients have great potential to incorporate α-TOC and other lipophilic bioactive compounds in various products to fulfill their biological functions.

## Figures and Tables

**Table 1:** Release equations of nanoemulsion.

Model	Equation	Correlation Coefficient (r)
First order	ln(1 − Q) = −0.0049t − 0.1709	0.8323
Higuchi	Q = 0.0456t^1/2^ + 0.0527	0.9330
Hixcon Crowell	(1 − Q)^1/3^ = −0.0016t + 0.9499	0.8098
Nibergull	(1 − Q)^1/2^ = −0.0021t + 0.9183	0.8182
Weibull	lnln(1/(1 − Q)) = 0.536lnt − 2.7257	0.9629

*Q* = *M_t_*/*M*_∞_, where *M_t_* is the cumulative amount of drug released in time *t* and *M*_∞_ the total amount of drug released.
